# 

**Published:** 1879-06

**Authors:** 


CONTENTS.

| | Page |
|---|---|
| Body and Mind . . . . . . | 1 |
| Summering . . . . . . . . | 1 |
| Public School Outrages . . | 9 |
| Memories Ruined . . . . . | 16 |
| Throat Ail . . . . . . . . | 17 |
| Cold Feet . . . . . . . . | 26 |
| Health of New York . . . | 27 |
| The Dead Wife . . . . . . | 28 |
| Household Economy . . . . | 29 |
| Diseases of Children . . . | 30 |
| Constipation . . . . . . . | 32 |

